# Emesis in pregnancy – a qualitative study on trial recruitment failure from the EMPOWER internal pilot

**DOI:** 10.1186/s40814-022-01093-1

**Published:** 2022-07-14

**Authors:** Mabel Leng Sim Lie, Catherine McParlin, Elaine McColl, Ruth H. Graham, Stephen C. Robson

**Affiliations:** Newcastle University, Newcastle upon Tyne, UK

**Keywords:** Qualitative study, RCT, Pregnancy, Emesis, Trial recruitment

## Abstract

**Background:**

As part of the internal pilot of the EMPOWER trial investigating the second-line antiemetic therapies in severe emesis in pregnancy (https://www.isrctn.com/ISRCTN16924692), a qualitative study of women’s views was carried out, to improve our understanding of why women did, or did not, consent to participation in the trial. Interviews were also conducted with site research staff, to broaden our analysis and explore other factors affecting recruitment.

**Methods:**

The sample comprised women who accepted or declined trial participation (*n*=21) and site research staff (*n*=22). A structured topic guide was used, in four email interviews and 17 telephone interviews with women, and semi-structured telephone interviews were carried out with staff. Of the women interviewed, seven had declined trial participation, and of the staff interviewed, 16 were research midwives/research nurses and six were principal investigators. All transcripts were checked for accuracy, anonymised and entered into NVIVO12 for indexing and retrieval. Data was analysed using a reflexive thematic analytic approach. In total, 72 codes were generated from the thematic analysis, and 36 from each sample group.

**Results:**

Three key themes based on all the interviews were (a) the diversity of recruitment pathways and boundaries of care, (b) the impact of trial complexity on recruitment and staff morale and (c) the ethics of caring for a patient with emesis. Ethical issues discussed included the use of double dummy and time to treat, particularly those suffering severely from the effects of nausea and vomiting. To illustrate these themes, staff perspectives are given more prominence.

**Conclusions:**

The main reason the trial was stopped related to the high proportion of women ineligible for recruitment due to prior treatment with study drug(s) because of unanticipated changes in clinical practice. The qualitative results also demonstrate the impact of the trial on women and staff and highlight how the diversity of referral pathways, boundaries of care and the complexity of the trial and protocol resulted in additional barriers to successful trial recruitment. Qualitative work in pilot and feasibility studies of a clinical trial is recommended, to evaluate whether recruitment strategies remain viable in unanticipated contexts.

**Trial registration:**

Trial registration number ISRCTN16924692. Date: 08/01/2018

**Supplementary Information:**

The online version contains supplementary material available at 10.1186/s40814-022-01093-1.

## Background

Successful patient recruitment is critical for the conduct of randomised controlled trials (RCTs), but a large proportion of trials do not reach their recruitment target and have to extend the recruitment period or close prematurely [1]. The economic and policy implications vary but are significant [2]. Efforts have been made to identify factors to improve trial recruitment [1, 3], and qualitative research in conjunction with RCTs is increasingly used to understand barriers and facilitators to recruitment. However, an attempt to apply a standardised complex intervention to improve recruitment using qualitative methods in RCTs was found to be more challenging than expected [4, 5]. Clinical trials of investigative medicinal products (CTIMPs) are subject to legislative and regulatory frameworks, and so the additional complexity involved is a key contextual factor for recruitment. CTIMPs are situated in specific times and places and are subject to unpredictability and uncertainties. Qualitative interpretative methodology that acknowledges this open (rather than closed) systems context [6] means that the complexities contributing to trial success or failure can be better accounted for. One strategy to illuminate these complexities is a pre-trial feasibility study or pilot phase with integrated qualitative research and detailed recruitment planning [2, 7].

Patient recruitment is particularly challenging in trials involving pregnant women [8–12]. Previous research has reported that women are motivated to join a trial because of the prospect of an improved outcome for their baby [8, 12] or a desire to contribute to medical science [9]. Research has also identified the need to build trust with women [11] and to address uncertainty about scientific research [10]. In the context of caution about use of medicines in pregnancy, recruitment of pregnant women into CTIMPS has the additional challenge of managing the responsibility they feel for their unborn baby [13]. In our study, there was also women’s vulnerable physical and/or emotional state due to prolonged experiences of vomiting and nausea to consider [14, 15]. In a study about experiences of being asked to participate in research [16], pregnant women highlighted the importance of a personalised approach which recognised the appropriateness of timing and a consideration of their vulnerability at that point in time.

The EMPOWER clinical trial, i.e., Emesis in Pregnancy—Ondansetron with metoclopramide, was commissioned by the UK National Institute for Health Research (NIHR) and incorporated an internal pilot phase to ascertain feasibility of recruitment to time and target. The population studied was women with severe nausea and vomiting in pregnancy (NVP) who had failed the first-line antiemetic therapy. The primary study objective was to determine which second-line hospital-prescribed therapy, in addition to IV rehydration, should be adopted as mainstream provision in the UK NHS. The trial design, as specified by the funding body, was a multi-centre, double dummy, double masked, controlled factorial design, to determine which (if any) second-line therapy (ondansetron or metoclopramide) reduced the rate of antiemetic treatment failure up to 10 days after treatment initiation. The 2016 Royal College of Obstetrics and Gynaecologists’ (RCOG) guideline [17] identified the trial study drugs as second-line treatments, to be administered only after the first-line treatments had failed. Nevertheless, patient and public involvement (PPI) advisors expressed concern about the double dummy design and the fact that one quarter of the study sample would receive no active drug. Therefore, as part of the EMPOWER pilot, an evaluation of trial recruitment included women’s views of the trial design with the aim of understanding why women did, or did not, consent to participation in the study.

The trial was opened to recruitment in April 2018, and the first woman was recruited in June 2018. However, trial recruitment progressed very slowly, and recruitment to the qualitative study was also slow. By the end of October 2018, when 17 women had been recruited, the protocol was amended to broaden the inclusion criteria and allow women who had been prescribed oral study drug prior to presentation to be included. This amendment was introduced, with support from the Trial Steering Group and the funder, when it became clear that many women were ineligible because of prior prescription of study drugs by general practitioners or Emergency Department (ED) staff. However, this did not improve recruitment to the trial. As a result, there was limited availability for interview of women who had consented to or declined trial participation. To help further understand barriers to trial recruitment, interviews were extended to include site research staff, in order to explore in greater depth, the perspectives of health professionals on barriers to and enablers of trial recruitment.

## Methods

The practical framework employed in our methods is firstly, that it is data-driven or inductive, and therefore descriptive, to answer ‘what happened’. It is then interpretive to address the ‘why’ questions, and reflexive, with constant comparisons across the two sets of interviews and reviews by research team members. We adopted a blended approach of qualitative methods, which can broadly be described under the umbrella of ‘reflexive thematic analyses’ [18]. Drawing on social constructivist methodology, we explored trial recruitment as a contextualised and interactive social process. The lead author’s position as a non-clinician interviewer, combined with the contributions of clinical and sociology colleagues contributed to rigour in the analytical process.

### Data collection

#### Participant recruitment

Women who had been screened and found to be eligible and had consented to or declined EMPOWER trial participation were approached for recruitment to the qualitative sub-study. Potential recruits were given a study information sheet and an expression of interest (EoI) form. Mindful that patients were suffering from severe NVP, attempts to contact women who had indicated willingness to take part were confined to between 14 and 28 days after the initial approach to participate in the trial. Telephone interviews were conducted with women who returned the EoI form and were contactable. Oral consent was obtained over the telephone, recorded and transcribed. A copy of the consent form filled in by the researcher on the participant’s behalf was sent to them by email or by post according to their preference. Women were also given the option of an asynchronous email interview if they felt too sick to hold a synchronous conversation with the interviewer at a specific time. They filled in a consent form by email attachment.

Research staff contact details were accessed via the Newcastle Clinical Trials Unit. The information sheet and consent form were e-mailed to research nurses/midwives and principal investigators (PIs) who had screened or recruited women to the trial. Those interested in participating returned the consent form by email or agreed to consent over the telephone.

#### Sample description

A total of 42 EoI forms (from 34% of all eligible pregnant women) were received. Between May 2018 and August 2019, 21 of the 42 women (50%) were interviewed, of whom seven had declined participation in the trial (see Table 1). Seven women (two decliners) were interviewed after the protocol amendment. Interviews with women lasted a median of 8.2 min (range 5.4–16.2). Four interviews took place via email.

**Table 1:** Interview study participants and non-participants

SITE	EoIs	Acceptors	Decliners	INTERVIEWED	Acceptors	Decliners
**Newcastle**	**9**	4	5	**4**	2	2
**Sunderland**	**10**	9	1	**4**	4	0
**Leeds**	**6**	3	3	**2**	1	1
**Birmingham**	**3**	1	2	**2**	0	2
**London**	**4**	4	0	**4**	4	0
**Bradford**	**5**	1	4	**2**	0	2
**South Tees**	**4**	4	0	**3**	3	0
**Portsmouth**	**1**	0	1	**0**	0	0
**Totals**	**42**	26	16	**21**	14	7

Women who had returned an EoI were often not contactable, even out-of-hours and at weekends. In some cases, repeat attempts to make contact were successful, but these were limited in number, due to the need to be sensitive to the women’s circumstances. Most of the 21 women who were not interviewed did not respond to the researcher follow-up contact (between 14 and 28 days after initial contact). Two women agreed to be interviewed but did not respond subsequently, while two women declined to be interviewed when contacted.

Most of the site research staff contacted were willing to participate; 22 research midwives or nurses (*n*=16), and PIs (*n*=6) were interviewed between December 2018 and September 2019. The majority (*n*=16) were interviewed after the protocol was amended, including all six of the interviews with site PIs, which were conducted towards the end of recruitment to the qualitative study. The telephone interviews lasted a median of 24.3 min (range: 12.1–44.5).

#### Interview methods

Ten of the 21 patient participants were interviewed over the telephone at their first point of contact, seven arranged a separate time for their interview and four women completed the interview through an exchange of emails. To keep to the short period of time assigned for the interview, the topic guide for the interviews was made up of a grid of questions and possible answers, to aid quick adaptation of the interview to the specific experiences of the participant and allowing each participant’s priority topics to shape the conversations. The interview allowed opportunities for women to expand on their answers wherever possible, about positive and negative reasons for participating. The main questions in the grid were as follows:

1. What did you think about when you were first asked to take part in the study?
2. Why did you / didn’t you want to take part in the study?
3. Did the way in which you were invited to take part affect your decision?
4. Was there anything about the study itself that made you want/not want to take part?

Of the trial research staff approached at each site, at least two research nurses/midwives and the PI (except one) were interviewed. Interviews took place by telephone and covered the following topics: experiences of trial set up and recruitment, difficulties and progress through the trial and suggested improvements.

In the case of the site research staff, interviews were less time-limited, enabling a more constructionist informed approach during the data collection phase to be adopted [19], but the topics covered were still similar enough to the interviews with women to allow both sets of interview data to be combined in a reflexive thematic analysis [18]. Drawing on the features of being inductive, comparative, interactive and iterative during data collection and on-going analysis, this meant that while a topic guide was followed in initial interviews, this was adapted in line with emerging findings requiring further exploration. A second topic guide was then designed based on these findings for the trial site PIs to describe their experiences of trial management oversight at their site. The following additional topics were explored in later interviews with site research staff, resulting in a richer corpus of data for analysis.

1. Proportion of women referred from ED
2. Attempts to raise the profile of the study with local general practitioners
3. Effect of the protocol amendment on recruitment
4. Impact of women with language difficulties or taking an anti-depressant
5. Involvement of research staff in the care of patients being recruited
6. Awareness that study drugs were being used in primary care

An opportunity for triangulation between the patient data and the clinician data arose, as staff described examples of cases of screening and recruitment that could be of interest to the study. Therefore, though recruitment to the patient group was more limited than originally anticipated, overall data saturation was reached, i.e., the data obtained allowed us to approach the point where no issues needed further confirmation or exploration, and no new themes emerged from analysis of either the patient or the site research staff interviews. Audio files of all interviews, files of the associated transcripts and of the four email interviews, were stored on a password-secure server. The transcripts from all interviews were checked for accuracy, anonymised and entered into qualitative data management software NVIVO12 for indexing and retrieval. Using the software, cases were created of the eight trial sites with interviews from pregnant women and research site staff grouped together for cross-checking. The presentation of participant data linked to site characteristics was, however, discounted, because of the risk of participants being identifiable.

The qualitative researchers were separate to the clinical research team members. As a matter of course, clinical members of the research team did not undertake interviews, and they did not have access to the transcript data until it was anonymised. Access to data was primarily via presentation of coded data grouped by theme, and access to full anonymised transcripts was kept to the minimum required to allow the clinical team members to engage meaningfully in the process of sense checking the emerging coding frame and themes.

### Qualitative data analysis

A blend of approaches was utilised to analyse the data in a systematic way as findings emerged from the ongoing iterative process. The following table summarises the stages that resulted as team members grappled with the significance of the findings [See Table 2].

**Table 2:** Stages in data collection and analysis

Stages	Approaches	Outcomes
Data collection and analysis	Reflexive thematic analysis	Revised questions and topic guide
1st team meeting	Reflexive thematic analysis	Review and revision of themes
2nd team meeting	Framework analysis and knowledge translation	Comparison of staff and patient data; charting under ‘hurdles’ and ‘enablers’
3rd team meeting	Narrative presentation of data from framework analysis with illustrative quotes	Qualitative report for funders
Publication	Conceptual themes based on the literature, drawing together the results from the reflexive thematic analysis and the framework comparative analysis.	Paper for academic journal

**Fig. 1:**
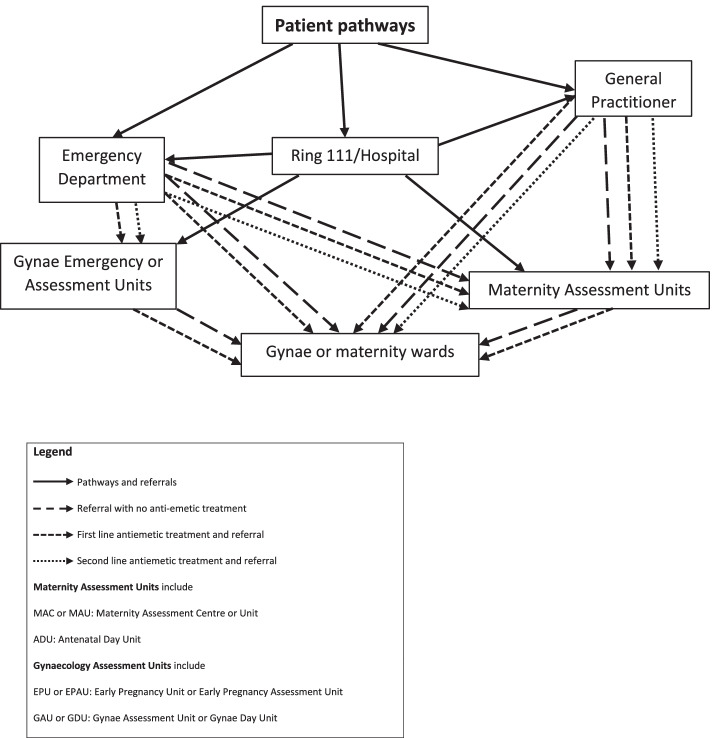
Referral pathways for women and sites for antiemetic prescribing

Overall, the data were mainly analysed using a reflexive thematic approach [18] in which codes were developed inductively from the reading and re-reading of transcripts, to form an initial coding framework that was significantly revised, expanded or collapsed, as the analysis progressed. The first four stages described by Braun and Clarke [20] provided a useful framework for describing the process the team followed in enhancing reflexivity and analytic rigour:

1. Familiarisation with the data: reading and re-reading of a sample of transcripts to identify and agree descriptive codes (ML and RG—one meeting on pregnant women’s data, and another on research site staff’s data)
2. Coding the data: coding the data according to an initial coding frame and revising the coding frame as further interviews were analysed, drawing on some of the techniques associated with grounded theory approaches such as line by line coding and constant comparison, aimed at uncovering social processes [18] (see Fig. 1). (ML)
3. Searching for themes: analysis of text coded in descriptive codes to determine at an interpretive level the themes that would best capture the findings from the data (ML)
4. Reviewing the themes: data analysis team meetings to review possible themes with the clinician chief investigator (SCR) and medical sociologist co-investigator (RG).

At the second data analysis team meeting, to enhance accessibility for a more clinically engaged audience and the impact of our findings in the clinic, two broad themes were agreed for further analysis, i.e., ‘hurdles’ and ‘enablers’ to recruitment. Considering the complexity of the data with respect to the differences in the sample groups, it was decided to take the data management approach used in framework analysis [16] and present the data in charts under the following groups: (1) women who accepted participation, (2) women who declined participation, (3) research midwives/nurses, and (4) PIs. This enabled the triangulation between the different participant perspectives within the data set overall.

In the third data analysis team meeting, the qualitative findings related to the two broad themes of ‘hurdles’ and ‘enablers’ were presented using illustrative quotes in a spreadsheet. A decision was made to present the key themes in the data, with selected quotes from the spreadsheet, in the text itself as in a standard qualitative report. This allowed a clearer link between the discussion of the themes and the available evidence from the qualitative data. Supplementary themes were identified but are not reported here. Further details are available in the trial report [21]. The final stage was an additional critically informed, interpretative strand of analysis, wherein the qualitative data were presented according to sociological, concept-led themes drawn from the literature, which are discussed in more detail below. In this way, the analytic approach therefore utilised both descriptive and interpretive perspectives and drew on a framework approach to pull these elements together into an analysis that was enriched through the reflexive movement between these perspectives as the substantive themes were generated.

## Results

Altogether 72 codes were generated from the NVIVO coding, of which 36 related to the interviews with pregnant women and 36 to those with research staff (see Additional file 1). The findings from these codes formed the framework describing the hurdles and enablers in the process of conducting trial recruitment (see Additional File 2). Three broad analytic themes were identified: (a) recruitment pathways and boundaries of care, (b) trial complexity and (c) the ethics of caring for a patient with severe NVP. Within these themes, research staff interviews were more prominent because with their broader experience and understanding of these issues, and they were better able to describe the complex contexts in which recruitment processes exist. Staff identifiers begin with ‘TS’ and women’s identifiers with ‘EMP’. The results move from the clinical context, describing the diversity of referral pathways and boundaries of care, to a closer focus on trial complexity and its impact on recruitment. The findings end with a consideration of the ethics of recruiting and caring for potential trial participants with NVP.

### The diversity of referral pathways and boundaries of care

There were multiple referral pathways (see Fig. 1) for women with severe NVP, depending, for example, on their gestational age, previous history of NVP and their knowledge of how to access care relevant to NVP.

Trial inclusion criteria stipulated women needed to have been treated with a first-line antiemetic for at least 24 h with no sustained improvement in symptoms before they could be approached about the trial. Most of the women were found to have had prior treatment with the study drugs (2nd line treatment), thus rendering them ineligible.

The complexity of pathways and varied times at which women presented made it difficult for research staff to approach women for recruitment before they were prescribed antiemetics. We had assumed that most women would present at maternity or gynaecological Assessment Units (AUs) or Day Units (DUs) in the first instance. In practice, women frequently presented out-of-hours, at weekends or bank holidays, often because they could not find the time during the week when they were at work or had family commitments. Research staff reported that women might also have had to wait until evening time for transport to the hospital by their partners who were at work. Most AUs and DUs were closed out-of-hours, so some women ended up at an ED. Others who presented at the weekend would often have a weekday follow-up appointment at a specialist AU.

The two key points along the pathways at which women were likely to be treated with a study drug were at their General Practice (GP) surgery or at an ED (Fig. 1).

Depending on the woman’s condition and circumstances, some GPs treated women with anti-emetics, while others asked them to attend the ED or referred them to hospital maternity or gynaecological services where they could be treated with intravenous fluids. Some women went directly to an ED or the AU themselves. Ethnic minority patients were perceived to be more likely to go direct to an ED.

We’ve got quite a large Eastern European population as well who don’t always you know, haven’t always been here that long and don’t always have a massive grasp of the language so sometimes their comprehension of where they need to go is not always, you know the same as somebody that might be able to speak the language and you know had pregnancies before and knows how it works. ‘Cause generally with everything else if you’ve got an issue you go straight to A&E don’t you? …. TS20

If women could only attend out-of-hours, they typically presented at AUs that had longer opening or weekend hours. However, research nurses and midwives were often not available out-of-hours and the clinical staff working out-of-hours were frequently unaware of the study or found it too difficult or time-consuming for recruitment to take place. Thus, the complexity of clinical care pathways presented a problem for research staff, as summed up in the following quote:

…inevitably some will come through A&E, others will come from the Early Pregnancy Unit, others will come direct from the GP, others will self-present. So there’s a wide range of stakeholders who will refer in women that of course they’ll self-refer as well. - TS18.

Recruitment was also affected by local hospital guidelines. Recruitment was more successful when the local policy in the ED was to transfer patients with NVP directly to specialised AUs as was the case at the following site.

Many were coming through day unit only because at the time our A&E was also moving, pushing us to move the patients from A&E as soon as possible either to our day unit or gynae ward. So it sort of helped us we said okay, that helped us recruit better as well so we said okay if you just give fluids and move them wherever like day unit or gynae ward and then we thought we can recruit and give the medication you know the trial medication. So it worked fine for us. - TS17

Care pathways for pregnant women also differed from hospital to hospital depending on the gestational age at presentation. In most hospitals, they were signposted from an ED to an AU but this could depend on gestational criteria. Alternatively, women were given an initial assessment and care plan at an ED. This influenced when women were referred to specialist AUs where they could be approached by research staff. In some hospitals, to ease the workload at MAUs, women with pregnancies at earlier gestation were seen within the gynaecology department (either in a GAU or on a gynaecology ward). Where a change in care pathway occurred during the course of the trial, this made identifying eligible women more complicated, as research staff would have to engage different colleagues in the recruitment of patients.

Prescribing policies in the hospital could be another hurdle to recruitment. Despite local PIs reporting that unit guidelines complied with national (RCOG) guidelines regarding the sequential use of anti-emetic drugs, in some units, study drugs were being administered as first-line treatment or multiple anti-emetics were being prescribed simultaneously. Staff at one site reported their usual practice was for cyclizine to be given with ondansetron and metoclopramide.

…our policy here is to give cyclizine and ondansetron with PRN metoclopramide so we’ve been trying to see if we can just give them cyclizine only - TS05

Staff therefore had to adapt local policy to accommodate EMPOWER. According to some research staff, they were not able to ascertain which antiemetic drug had been prescribed in the community because they could not access GP electronic records and women were unsure of prior medication use. Many women had not yet formally booked in for antenatal care and community midwives were therefore not involved in their care. A comprehensive screening log and an electronic record system allowed some staff to track where patients had come from and to look at their care plan and prescription records. However, if they were transferred from another unit to which the electronic systems were not linked, this became more difficult. As a result, research staff often had to speak to the patient directly to clarify prior drug use, adding extra time and effort.

Thus, the boundaries between EDs and specialist AUs, as well as primary and secondary care, out-patients and in-patients, maternity and gynaecology departments, became pertinent. Successful recruitment depended on the goodwill and working relationships between staff within and across different service areas, as the following sections illustrate.

#### Working to ‘catch’ women at the right time on their care pathway

From staff accounts, in addition to unpredictability of presentation, infrequency of presentation was also another hurdle to overcome.

I think with EMPOWER we found ourselves running a study in which the participants, the participant’s attendance at a particular point in the pathway where recruitment was taking place was relatively infrequent. And I think that made it difficult for people to, for the clinical staff to always be on the ground, and always be on the ball if you like – TS18

when I’ve worked on studies we’ve had women booked into clinic ……..this way you know, we don’t know who’s coming through the door. So I would say it’s probably one of the most difficult ones that I’ve worked on but just in terms of catching the women and, you know, finding the ones that are eligible so. - TS01

Research staff worked hard to approach women at the most opportune time along the various pathways. To do this, the work of getting clinical colleagues engaged in the study was difficult but crucial.

Clinical colleagues needed encouragement to follow the RCOG step-up antiemetic guidelines, and (for medical staff) in checking eligibility, taking consent and prescribing the trial drugs.

…we were trying to drill it in and at every opportunity we were saying who, and we were sending emails if they’ve been given, randomly checking in A&E and the antiemetic. We were sending an email saying that they were to strictly follow the step-up regime - TS17

As part of trial governance, a regularly updated delegation log is kept, recording research site staff and their identified tasks with authorisation from the site principal investigator confirming their competency to carry out their duties. This included Good Clinical Practice (GCP) training obtained within 2 years of practising which was sometimes seen as an added burden to already busy workloads. Some PIs were very active in engaging clinical colleagues by running training sessions to raise awareness about the trial, whereas others were less so, depending on their lead research midwives to do so. Ongoing reminders to keep staff on board helped facilitate recruitment.

They’ve all got our telephone numbers as well as the number to all the research team so if anybody comes in that’s kind of eligible, they’ve got the eligibility criteria drilled in to them and there’s posters everywhere, so if they need to they’ll give us a call and then I’ll go over just have a look in the lady’s notes just to make sure that she definitely is kind of ticking all the boxes. - TS05

While research staff could take some time to become familiar with trial procedures, clinical colleagues also required time, with some getting to be ‘very good and they’re very used to it now’ (TS13). Nevertheless, when recruitment rates did not meet expectations, most of the work of research staff involved keeping the profile of the trial ever present in the minds of colleagues. Thus, as the pilot trial progressed it was felt less likely that eligible women were missed. Nevertheless, as research staff remained very mindful that they might miss the chance of recruiting a patient, they constantly visited relevant clinical areas to try and reach eligible women before they left hospital.

So I try and catch them as soon as I can because if you leave it later then there’s the risk that they might have already been given some medication….. And if I went down and there was somebody on the ward to come I would just sort of keep popping up and down and like I say try and catch them as soon as they come in – TS01

….like if I work on a day shift then I would go to the day unit maybe about three or four times to look for…. to check the ladies with hyperemesis [ ] because they come in and they can be in for an hour be re-hydrated and then go home, so – TS10

Once clinical staff within the maternity and/or gynaecological departments were on board with the study, screening and recruitment took place even among in-patients.

I think it depends on the way that your Trust is set up so we, we have the obs med team and they go and see the ward every single day to see if anyone’s been admitted with hyperemesis. So those would be the very poorly ones. – TS08

Where research and clinical roles were very clearly defined, it was found that this could affect recruitment and patient care negatively. AUs operated differently according to whether women were considered as out-patients or in-patients. The following quote suggests an out-patient model of care and less role differentiation between research and clinical roles could have worked better for recruitment to EMPOWER.

….if you had say a model of care which looked after these women pretty much as outpatients or almost as outpatients and so you had quite a tight core of staff who were involved in that style of care, I could see the possibility of those individuals being the ones who recruited and signed up and looked after and what have …– TS18

Thus, seamless flows across departmental boundaries of out-patients and in-patients and/or maternity and gynaecology made ‘catching’ patients at an appropriate time for recruitment much easier.

#### Working across professional boundaries

There were many accounts of good relationships and rapport between staff demonstrating how personal relationships can mitigate against the institutional protocols of practice and role differentiation. Because of the variation in both the referral and management pathways available to women, it was often necessary for research staff to liaise with colleagues in different clinical areas, especially between maternity and gynaecology. Having to depend on colleagues, especially doctors in a busy department, created additional challenges for research staff.

Once a potential research participant was identified, responsibility for her care was restricted to unit or ward staff, with research staff only lending a minimal amount of help. However, in one busy AU, research staff contributed to the care of eligible women while they were considering the trial.

….what I’ve tended to do is not just give the information sheet and come away but I’ve usually kind of helped with starting IV fluids and you know making sure that if the patient has any questions that I’m there to answer, you know - TS04

Good relations were fostered when research staff understood the pressures that clinical colleagues were under, and the added burden placed upon them by participation in trials and subsequent recruitment activities.

*…they’re working flat out and we’re constantly asking them to remember something new and something extra and doing extra jobs. So the more you can kind of give back and take off them in terms of their workload then yeah like you say the better the relations between the two are. – TS20*

In other sites, clinical staff were seconded to research part-time. This was felt to work better than external staff doing research jobs.

*Yeah we’ve found, we’ve tried both, we’ve had external people come and just do research jobs and we’ve done the secondments and the secondments work so much better for a number of reasons, so we’ve stuck to that as a model now. – TS20*

This model of joint roles facilitated the negotiation of boundaries between the clinical and research work performed by nurses and midwives. However, one site found that the role of research staff could not be replaced by clinical staff out-of-hours.

*I thought we could involve them in recruitment out-of-hours but still that would not solve the problem because of the lot of history sheets, sheets needed to be filled out at the time of recruitment…. Only the research nurses were very well clued up with this study only could fill them -TS17*

After the research nurses/midwives had done the initial work of recruitment, there was work required of other colleagues. For example, engagement with pharmacy was another professional boundary that needed to be negotiated:

*…we had to get the drug from there [Ward x], make sure that the temperature of the fridge was adequately controlled and pharmacy were involved and pharmacy did conduct regular checks – TS22*

Unfortunately, at least one patient could not be recruited from one unit at a trial site with different arrangements, because there was no one from pharmacy available to dispense the medication when needed.

### Trial complexity and its impact on recruitment progress and staff morale

When research site staff were asked how EMPOWER compared with other trials they had worked on, complexity was a recurrent theme in many responses. The trial management team were aware of potential complexities presented by the double dummy, double masked, controlled factorial design imposed by the commissioning brief and were therefore careful in their site selection to include those with experience of conducting CTIMPS in pregnancy.

How the demands of the CTIMP affected the progress of research site staff in recruitment was variable. For example, it impacted on the willingness of clinical colleagues to assist with the trial, especially given the time involved in caring for very sick patients.

*… we have had success with other clinical trials which haven’t been quite as involved as EMPOWER and the clinical staff have helped with the recruitment to the trials…….it’s a little bit more time consuming that clinical staff aren’t able to fit that in with their clinical, you know, their needs really, the time that they have available. – TS02*

Although the site may have had a track record of recruiting to CTIMPs, not all research site staff had the same level of experience at the time of EMPOWER. The difference between a CTIMP and non-CTIMP was noted by the following research nurse, who was less experienced in CTIMPs.

*I do studies that aren’t CTIMP so I see the difference between the ones where there are drugs involved and how much more adherent they are to the rules and regulations which is totally understandable….I find the database not easy to use….We have had to call [the clinical trials unit] to clarify on every single occasion – TS02*

The following quote describes how difficult it was for research staff to explain the four arms of the trial.

*I think it’s quite a complex study to explain to patients….I think it’s complex when you’re trying to talk to a patient when it’s a randomised controlled trial and there’s only two groups, but wait, you’re talking about there’s four different possibilities – TS10*

The amount of information to take in was described by the following woman.

*A lot of information yeah, it’s a lot of information. But luckily everybody’s been really supportive and just you know there was, there were a few people who came through and just discussed everything with me to make sure that I knew what was happening… just to make sure I was not delirious [laugh] because I was very dehydrated by the time they got to me - EMP1702*

Procedural issues on eligibility and data input involving separate databases for randomisation were not straightforward and required contact with the clinical trials unit if research staff at site were unsure of what to do. Even with support, trial complexity had an impact on staff confidence and morale.

*….it sounds awful but you kind of dread actually recruiting someone because you know it’s so complicated – TS08*

On top of procedural complexities, speaking to women with NVP required more effort, according to the following staff member, more so than in other clinical scenarios, even in maternity.

*I would even say that like we’ve done studies on labour ward where you’re speaking to ladies in labour and sometimes that’s been easier than trying to speak to a lady who’s puking her guts up on the, on the assessment unit – TS10*

While most women interviewed were satisfied with the information provided, some referred to being too unwell to register everything in detail.

*I think it was, I think it was two pages, which is probably, I mean to be honest I was actually quite out of it because I hadn’t erm, I just had a really awful night of not sleeping - EMP1702*

#### Staff morale

At the start of the trial, research site staff were feeling motivated about the trial but as it progressed, some interviews suggested that their frustration started to affect morale when they found that they could not recruit the participant numbers needed.

*It’s difficult and just to keep the momentum going actually you doing a great job because you are going up every day and you’re screening. It’s just that we’re just not successful in recruiting. - TS14*

Some research staff noted that when they did manage to recruit women to the trial, there was a real sense of achievement, which could be linked to a lot of effort and teamwork.

*…. we were kind of getting a little bit, that we keep on looking and we can’t find women that are fitting the criteria but having the recent two recruits has been a real boost – TS06*

*The other day we did get the woman in the study there were three members of the research team that stayed two hours late and one of the consultants who stayed an hour over to finally get somebody in. So we certainly feel like we’ve gone above and beyond …- TS20*

However, the desperation to get a recruit could translate to being over-keen to persuade a woman to consider the trial.

*But it was kind of, felt as though I’m waiting for some treatment which I’m not getting but the research information was more important, it felt to me, ‘cause I had got a visit about three times. - EMP1401*

When women were not recruited ‘*every single day’* (TS14), there was evidence to suggest that this affected or diminished the enthusiasm that had been evident at the start of the trial. In one example, a research staff member described her frustration when after a long spell, a patient could not be recruited due to a database problem and trial management staff were unavailable out-of-hours.

Research PIs often boosted morale by making comparisons with other EMPOWER trial sites and reminding their staff of the recruitment difficulties faced by other trials more generally. Staff with more experience of trials seemed more resilient, and less deflated than newer research staff. For some, the challenge only strengthened their determination, especially if the site has had a good record of recruiting to time and target in the past—‘*we will get you one, I promise*’ (TS12).

### The ethics of recruiting (and caring for) potential participants with severe NVP

#### The chance of a double dummy

Concerns about recruiting women with severe NVP to the double dummy (placebo) arm, required by the funders, were raised during discussions, especially with PPI groups, in the design of the trial, but attempts were made to address them by incorporating the 12-h review to assess responsiveness to study drugs.

*….as a part of the study it was deemed that these women could come off it if there was no improvement…. So if after twelve hours their symptom hadn’t improved, so, yes there was a challenge, ethical challenge but there was an answer to that challenge as well – TS22*

However, this review did not conform with routine clinical management timelines in specific settings.

*……it’s explained you know that after, they’ll re-assess the situation after twelve hours and if they’re no better they’ll go on to something else. But I suppose it was one of those studies that you think there’s, you know the clinical management who wouldn’t normally do that -TS15*

As some women were unwilling to subject themselves to the possibility of being given no antiemetic treatment for 12 h—particularly if the double dummy arm was interpreted as being ‘no treatment’—careful reassurance about rehydration as therapy and their care during 12 h was essential.

When research staff were explaining about participation in the trial, they felt that reassurances the woman would be taken care of during the trial were important.

*I always make sure that they realise that if their sickness doesn’t improve that we won’t just, you know say no, no you’re in the trial you’ve got to continue. I do say that we will listen to them and that we will, you know make sure that, that they then have appropriate treatment if what they’re having isn’t appropriate for them - TS02*

#### Patient care and comfort

Research site staff expected that, apart from a short wait at the beginning for research staff to arrive and go through the recruitment process, the routine care of women with NVP would not be disrupted to any great extent.

However, there were reports that at times, the severity of the symptoms and workload pressures required that routine care took precedence over the trial.

*… they’d want us to go sort of there and then and if the woman was like, oh no I feel too poorly, then the doctor would just be coming to give them something and sending them on their way after the fluids are finished. – TS03*

In a less pressured context, the recruitment of patients with NVP would start with the admitting midwife, who would present the trial to the patient, assuring their care, and paving the way for the research midwife to approach the patient. Care and consideration for the patient were also evident in how and where women were approached about the trial when they were waiting to be seen by the doctor. In some sites, patients were in waiting rooms with other women, while in other sites, there were dedicated areas or side rooms where women could rest on armchairs or recliners in a more private space where they were rehydrated while waiting.

Some women were taken to a room or four-bedded bay quite quickly after triage. Staff reported that when women were made comfortable, they were more willing to read the information as it gave them something to take their mind off their condition, unless they were too ill to even do this. Women who were feeling comfortable were more amenable to being approached.

*…there were a couple of things that they asked us if I wanted to do. I said yeah I was just sitting there, on a drip, being rehydrated so, thought may as well – EMP1001*

Recruitment to the trial involved time, empathy and patient care. Time was needed for the woman to consider the information carefully before making an informed decision about consenting to participate or not.

*….the care in the early pregnancy unit was fantastic and then when I agreed to take part in the trial, there was the sort of, midwife, and then there were a few people there, one lady who kept making sure that I was okay, just coming in chatting to me and making sure I was fully understood and if they thought I hadn’t read one of the consent forms which I hadn’t they came back and sort of double checked and made sure that I understood what was needed to be signed and what not, so, it was very good – EMP1703*

At the same time, pregnant women needed to know that their care was not secondary to the research nor was it being held up unduly by the requirements of the research. Staff suggested that showing concern and keeping women informed all along their care pathway helped the situation.

#### Patient care and staff conscience

Research staff expressed their motivation to conduct the trial because they empathised with women and recognised the importance of the study.

*But hyperemesis is quite a debilitating condition, and I think a lot more research needs to be done in it. So I was interested to see, you know women’s thoughts on it and you know, hopefully that we could, you know in the long run improve the care that women get with hyperemesis. - TS21*

However, empathy could also have the subtle effect of raising ambivalent feelings about recruiting particular women who research site staff felt were at the limit of what they could cope with. Putting them through additional research requirements, such as a longer wait (to read the information and for the doctor to consent and prescribe) was suggested in some interviews as being a step too far. Some staff expressed strong empathy with women’s social circumstances and wellbeing, and a trial involving a placebo was viewed as too demanding on certain individuals.

*… people have got to survive on something haven’t they and pay their mortgages or their rent, you know, and look after their families so you know. And some people are just feeling that awful they want to know they’re having medication they don’t want to know that they’re, you know having a placebo. So you can appreciate that, I can ‘cause I’ve had hyperemesis myself – TS09*

Women whose vomiting did not improve could also affect the consciences of research staff. While women were reassured that they could end their participation if their symptoms continued, a midwife became concerned about an individual who wanted to continue with the trial even though she was feeling so poorly. To her, the measures used to assess symptom severity were not enough to capture the full impact of NVP. Some concerns about the measures used to assess women’s emotional states were expressed by some staff, as well as by one pregnant woman. This speaks to the limitations of standardized clinical measures in truly capturing lived experiences.

*…..two of them have scored above the trigger score for us but it’s circumstantial it’s not how they really feel about their everyday life, so. – TS02*

*You know the depression scale because it is, it’s how you feel at the moment. Like even when I’ve been to see the GP they’re obviously asking you are you okay and things like that and I get upset when they ask me am I okay. But I am okay but it’s, I’m not okay at the time if you know what I mean? - EMP1004*

Staff were well aware that while they empathised with the women and their experiences, and were concerned about their wellbeing, they had to exercise care in how they supported women during the trial.

*Sometimes the women are just under that [PUQE score] but they’re feeling absolutely dreadful but, they’re feeling like they can’t stop the trial because they’ve got ten days’ worth of treatment….. it’s quite a difficult situation to handle because I’m trying not to exert any forces that are going to create bias in the trial. But I am saying to the women if this treatment isn’t working for you, you need to say because we don’t know which arm of the trial you’re on - TS02*

According to the midwife, this woman felt responsible for failing the trial rather than the ‘treatment’ failing her. From the quote above, and other feedback from interviews describing empathy for women with NVP, it appeared that research staff commitment to a trial could be compromised when it caused them to feel uncomfortable about the effect of the trial on patient wellbeing. This was also illustrated when a SUSAR was submitted because of a fetal abnormality detected on ultrasound, leading to termination of pregnancy. Research staff expressed distress and felt responsible for the woman’s treatment with the study drugs.

#### The need to know

Another ethical question concerned the tension between the double-blind element of the design and women’s desire to know what drugs they were on in the trial when their treatment was completed. The following women contemplated the consequences of having to manage without knowing what ‘treatment’ they had received.

*I think my only concern was that after ten days you wouldn’t know what it was if it did help….. … if it had have worked I wouldn’t want to be then ill again while I try and figure it out – EMP1004*

*It would have been useful to know when I decided to stop the trial as I was prescribed ondansetron from the ward and this may have been what I was already on - which wasn't working. - EMP1302*

As one site research midwife commented,

*It would be nice for the women to find out what they were on at the end, because obviously some of these women are really, really poorly before they go in and then if they finally find something that makes them feel better….. It does seem a little bit mean [laughs] not letting them know really ‘cause they could go back to sort of, they could go back to the start again then, and sort of be really poorly - TS03*

From other accounts, where women needed ongoing treatment, research staff were considerate in making sure that worried individuals were prescribed both the study drugs after the trial.

There were several examples where site research staff encouraged women who had completed their participation in the trial to approach their GP for a prescription or to come back to the hospital for further treatment. Some women also independently requested the trial drugs after 10 days of treatment in the trial.

*I was travelling [ ] the day after the trial ended so asked for both drugs to be given after the trial as I didn’t want to get sick whilst I was away. I may not need both but I wouldn’t know this until I find out (if I do) what medicines if any that I was on – EMP1002*

When one GP refused to prescribe one of the study drugs following trial participation, the research nurse (TS11) arranged for a prescription from one of the on-call hospital doctors. However, while initial concerns about un-blinding were dealt with through the initiatives of both the research midwives/nurses or the women themselves, this did not take away the desire for women to know what treatment they received after the trial was completed.

*I just think sort of ethically I would although you sign up to do a trial you do have a right to know what you took because now I’m guessing I was on Metoclopramide so in a way, or you know guessing that’s the best one for me to take erm yeah I would, I would like to know what I took. - EMP1702*

## Discussion

The EMPOWER trial was designed with an internal pilot phase to test key processes of the main trial—specifically recruitment. However, despite the best efforts of those involved, including a protocol amendment and an extended pilot phase, the required numbers of pregnant women were not recruited. The overall recruitment rate was 29% of eligible women and this ranged from 11 to 63% across different sites [21]. Patient recruitment is challenging in a CTIMP involving pregnant women with NVP. The qualitative interview study of women approached to participate in the EMPOWER trial was therefore based on the assumption that the success of the trial hinged mainly on their willingness to participate in the trial. While the reasons that women decline participation in the trial are described in more detail in the trial report to commissioners [21], this paper prioritises the findings from research site staff because of the more extensive and nuanced data collected from their interviews. The findings suggested that there were many other barriers, over and above women’s willingness, to successful recruitment. Apart from the characteristics of the study population, there were pertinent contextual and structural features of the trial itself. In particular, the culture of prescribing the study drugs in primary and secondary care varied from site to site and a large proportion of women with severe NVP were ineligible because they had already been treated with one of the study drugs [21]. A fully informed discussion of the reasons for this practice culture has been provided in the HTA trial report.

In this paper, we presented the findings that patient referral pathways were diverse and the presentation of women at study sites was both unpredictable and infrequent, with women often presenting out-of-hours when research staff were unavailable. Recruiting emergency care patients for RCTs during out-of-hours is a challenging task more generally [22, 23]. To reach target recruitment levels, site research staff therefore were challenged to negotiate departmental and professional boundaries of care, to approach eligible women at every available and suitable opportunity. The importance of the point at which individuals are approached to participate in a trial has also been highlighted in the OPEN trial [24].

The ‘micro-level processes of boundary work’ [25] not only apply when new professional roles are introduced into a healthcare system, but also between different established roles when new care pathways are implemented. Clinical interventions and changes in management are regular features in healthcare but boundary issues can also arise when a trial of any intervention is undertaken within a complex NHS department such as obstetrics and gynaecology which, in larger units, often have separate nursing and medical consultant workforces. Baldwin [26] refers to specialist midwives as having a role as ‘navigators’, providing continuity of care across departmental boundaries; our findings suggest the research midwives/nurses in the EMPOWER trial fulfilled a similar role. Working with fellow professionals to deliver trial processes was challenging because of the complexity of the EMPOWER trial protocol, especially with respect to procedural and administrative tasks. The difficulties that the research nurses or midwives in EMPOWER (like specialist nurses [27]) have in engaging doctors to carry out tasks beyond their already busy workloads are not new; in this case, the work required in screening, consenting and prescribing treatment was perceived by staff to be more demanding in comparison with many other trials. In addition, these doctors on shift were required to have had Good Clinical Practice training, although this has been reconsidered in the more recent guidance [28]. The findings revealed collaborative boundary work [29] occurring between obstetric and gynaecological departments to facilitate recruitment, but a slightly different process occurring in the care of patients where the boundary between research and clinical care was more distinct. The area of intra-professional boundaries has received less attention [27], in this case where research nurses or midwives have a different status to those more routinely administering clinical care. Organisational management of professional responsibilities, rather than competitiveness [29] appeared to be what reinforced role differentiation between clinical and research staff. In the context of this study, the ‘symbolic boundary’ [30, 31] between ‘research’ and ‘clinical’, while present, was negotiated differently at different sites; in some units, these roles were combined, with positive effects; in others, the role differentiation was maintained throughout. This study was unable to unpick the dynamics of the relationship between research and clinical staff in full but highlighted the key contribution that empathetic working relationships across these boundaries could have on trial recruitment. The analysis also identified other boundaries to be negotiated, between primary care, emergency care, hospital outpatient and in-patient services, and also specialist Women’s Services. These boundaries affected women’s screening, diagnostic and treatment pathways and hence recruitment.

The analysis identified that as result of trial complexity, there arose a number of motivational and ethical dilemmas for the staff involved. The strain of not achieving recruitment success impacted on staff morale and determination. In addition, the slow rate of recruitment meant that remembering all the processes relating to recruiting a participant could be challenging for staff, particularly those who were less experienced. Staff perceived ethical challenges in consenting physically debilitated women to a trial where they might receive a double dummy and where recruitment might incur delays with antiemetic therapy. Staff who provide care for women have an obligation or duty of care to treat the women as soon as they can, rather than for both clinician and patient to be delayed by trial processes and paperwork, especially in an emergency setting. As our findings demonstrate, patient-centred practice where research staff maintain their role as patient advocates may clash with the demands of the model of evidence-based medicine in clinical trials [32]. Nevertheless, experienced research nurses/midwives, and a supportive team approach [22] facilitated trial recruitment at several sites.

Given the constraints set out in the commissioning brief, the trial was designed with four arms, one with a double dummy. This raised ethical issues associated with women’s desire to know which arm they had been in to inform ongoing treatment beyond the trial. The challenges of recruiting patients into placebo-controlled trials are well-recognised [33, 34]. The PITCHES trial involving antenatal women [35] reported that of the 52% of eligible women who were screened, 57% declined, of whom 13% did not want to be randomly allocated. In this trial, treatment preferences were a factor as 14% did not want the treatment medication, but 35% did. The evidence of the impact of placebo arms in general surgery research is also mixed; one orthopaedic study reported a 60% decline rate [36], while a systematic review reported that surgical RCTs with a placebo arm are feasible for procedures with a lower level of invasiveness [33]. But, as in EMPOWER, the review did acknowledge that the main challenge in completing a trial was identifying sufficient numbers of eligible patients. Interviews with women approached to participate in EMPOWER also confirmed two divergent themes; an explicit reluctance to risk receiving a placebo versus an altruistic desire to help increase scientific knowledge [34]. From the patient perspective, pregnant women have been found to be motivated to enter a trial if it could lead to an improved pregnancy outcome [6, 10], but at the same time, they felt responsible for their unborn child [11].

Exclusion criteria may inhibit recruitment at the required rate [37] as in the case of EMPOWER where recent evidence suggests 38–59% of women admitted to hospital with severe NVP/HG have already been prescribed antiemetic drugs by their GP [38, 39]. But beyond this, our findings point to the importance of and need for feasibility and piloting work before a trial commences [2, 5] and the essential work of site-specific contextual understandings of the organisation of care, including a programme of measures to communicate the aims of the trial targeted to engage the interests of both clinicians and patients likely to be involved.

The study has a number of limitations. The number of interviews were restricted by the low recruitment of pregnant women into the trial. However, one third of the sample of women were decliners and they made up nearly half the group of women who declined participation and had returned an EoI. Interviews were also limited by women’s availability, and how well they were feeling. Research midwives/nurses were approached for interview based on the number of women they had screened or recruited, but they self-selected in volunteering to be interviewed. One PI was not contactable, but the lead research midwife at that site was interviewed. Although more staff members could have been interviewed because of the extension of the study to include four additional sites, very few women ended up being eligible at these later sites so staff had no or very limited experience of the recruitment process in practice. Further, given that no new themes were emerging from the original pilot site staff interviews, this led us to believe saturation had been reached. On balance however, interview data from research staff dominated the analysis for this paper.

There are a number of research implications from this work. The EMPOWER trial was designed in response to a commissioned call by the UK NIHR. The findings from this pilot and particularly the qualitative evaluation underscore the value of a pilot phase in any commissioned call for an RCT. Further evidence for this can be found in prior trials which did not progress from pilot to full trial [40, 41], or where trials were modified as a result of findings from the pilot [24, 42]. Our study findings reveal the need to understand the complexities of site-specific clinical practices vis-à-vis national and local guidelines. The evolving culture of prescribing anti-emetics both in primary and secondary care, within each trial site was a critical element that affected the numbers of eligible women.

## Conclusion

The progress of EMPOWER was not helped by the need for four arms (including a double dummy arm) and the need for medical confirmation of eligibility and consent. CTIMP requirements aside, the trial was complicated by the fact that the women were uncomfortably sick, and staff had to balance the volume of information they were required to impart to ensure ethically appropriate recruitment to the trial, with delays in women getting treatment. Nevertheless, these challenges were mitigated by the good relationships spanning departmental and professional boundaries.

The qualitative study was limited by the number of women interviewed during the pilot phase of the EMPOWER trial. However, the staff interviews uncovered insights into the trial we would never have reached if the study had not been adapted to include their perspectives. The importance of a holistic account of a research trial recruitment process, encompassing experiences from both patients and health professionals involved is underlined in this paper, as is the importance of the need for feasibility studies, and qualitative work in both feasibility and pilot stages of a clinical trial. This is because of the recognition that health systems, in which care pathways and clinical protocols are integral parts, are complex. To address the research-service gap, future studies should consider the approach taken by complexity theory [43] which takes into account issues such as unpredictability, interdependencies, adaptive capabilities and conflict, which have been highlighted in this study.

## Supplementary Information

**Additional file 1: Table S1.** Altogether 72 codes were generated from the NVIVO coding, of which 36 related to the interviews with pregnant women and 36 to those with research staff.

**Additional file 2: Table S2.** The findings from these codes formed the framework describing the hurdles and enablers in the process of conducting trial recruitment.

## Data Availability

The datasets used and/or analysed during the current study are available from the corresponding author on reasonable request. We aim to prepare and deposit the qualitative interview data in the UK Data Archive at the University of Essex.
