# Supplementary material for: Emesis in pregnancy – a qualitative study on trial recruitment failure from the EMPOWER internal pilot
Source: Pilot Feasibility Stud. 2022 Jul 14;8:146. doi: 10.1186/s40814-022-01093-1 (PMC9281005; doi:10.1186/s40814-022-01093-1)
Supplement: Supplementary file 1 — Additional file 1: Table S1. Altogether 72 codes were generated from the NVIVO coding, of which 36 related to the interviews with pregnant women and 36 to those with research staff. [file 40814_2022_1093_MOESM1_ESM.docx]

| **Name** | **Files** | **References** |
| --- | --- | --- |
| **PATIENTS** | 0 | 0 |
| Ability to engage with written info | 4 | 6 |
| Beliefs about medication | 7 | 8 |
| Benefits of unblinding | 7 | 8 |
| Comments on trial design and recruitment | 9 | 12 |
| Future participation | 10 | 10 |
| Manner of approach for recruitment | 12 | 15 |
| Patient journey and experience | 17 | 37 |
| The effect of sickness | 13 | 26 |
| Patient satisfaction, care and follow up | 12 | 21 |
| Previous experience of research | 12 | 15 |
| Reactions to trial blinding | 9 | 16 |
| Reasons for involvement | 0 | 0 |
| Assurance of drug safety | 5 | 7 |
| Help with one's own sickness | 13 | 17 |
| Helping other women | 4 | 6 |
| Need for medical research | 10 | 12 |
| Option to withdraw at any stage | 6 | 13 |
| Passive compliance, implicit trust | 3 | 5 |
| Persuaded by information presented | 1 | 3 |
| Reasons for non-involvement | 0 | 0 |
| Being off work | 1 | 1 |
| Burden of paperwork | 6 | 7 |
| Confidence in a known treatment | 4 | 6 |
| Family responsibilities | 5 | 8 |
| Hospital stay | 2 | 3 |
| IV fluids worked for her | 1 | 2 |
| No unblinding | 2 | 5 |
| Risk to pregnancy | 5 | 8 |
| The risk to self (double placebo) | 6 | 12 |
| Treatment delay re urgency | 2 | 8 |
| Recruitment miscommunication | 2 | 2 |
| Timing of approach for recruitment | 16 | 34 |
| Understanding trial information | 18 | 30 |
| Prior understanding through PIS | 1 | 1 |
| Understanding timescales | 1 | 2 |
| Would like study results | 4 | 4 |
| **STAFF** | 0 | 0 |
| Eligibility criteria | 16 | 38 |
| GP prescribing | 8 | 9 |
| Language issues | 6 | 6 |
| Engagement with CTU | 14 | 22 |
| End of trial | 4 | 4 |
| Improving trial processes | 19 | 43 |
| Unblinding | 1 | 1 |
| Location and logistics | 9 | 12 |
| Patient care | 19 | 37 |
| Clinical or research roles | 8 | 9 |
| Staff empathy | 4 | 5 |
| Patient examples and feedback | 16 | 41 |
| A welcome study | 6 | 10 |
| Inability to engage with the written | 8 | 12 |
| Patient treatment preference | 5 | 7 |
| Placebo avoidance | 6 | 10 |
| Post trial experiences | 7 | 7 |
| Requests for unblinding | 3 | 3 |
| Stoical women on trial | 2 | 4 |
| Perceptions of patient understanding | 14 | 24 |
| Progress and morale | 14 | 19 |
| Qualitative study recruitment | 7 | 7 |
| Recruitment or referral pathways | 20 | 45 |
| Screening and recruitment | 22 | 78 |
| Catch me if you can | 16 | 23 |
| Counselling | 2 | 6 |
| Patients being too unwell | 13 | 23 |
| Visual aids | 6 | 8 |
| Site setup and specific issues | 17 | 38 |
| Staff experience of trials | 22 | 35 |
| Staff interview study queries | 4 | 5 |
| Staff issues and buy-in | 20 | 57 |
| Study profile | 12 | 21 |
| Time issues | 19 | 44 |
| Trial organisation and complexity | 19 | 50 |
| Views about amended protocol | 17 | 22 |
