# Supplementary material for: Emesis in pregnancy – a qualitative study on trial recruitment failure from the EMPOWER internal pilot
Source: Pilot Feasibility Stud. 2022 Jul 14;8:146. doi: 10.1186/s40814-022-01093-1 (PMC9281005; doi:10.1186/s40814-022-01093-1)
Supplement: Supplementary file 2 — Additional file 2: Table S2. The findings from these codes formed the framework describing the hurdles and enablers in the process of conducting trial recruitment. [file 40814_2022_1093_MOESM2_ESM.docx]

Appendix

**Table 2: Hurdles to recruitment**

| **Topic** | **Hurdles** |
| --- | --- |
| **Requirements of the clinical trial** | Adherence to RCOG guidelines |
|  | 2nd line adopted as normal practice |
|  | Study question out of step with current practice, and not having a high enough profile with clinicians |
|  | Liaison between maternity and gynae needed extra work |
| **CTU** | Advice not available when required |
|  | Not available out of hours |
|  | Data entry error |
| **Demands of a CTIMP protocol** | Consent process takes time |
|  | GCP trained doctor needs to consent and prescribe |
|  | Waiting time for eligibility |
|  | 12 hours to see if the IMP really does not work |
| **Publicity and education about the trial** | GPs and community midwifery |
|  | A&E staff |
|  | Clinical staff on duty and out of hours |
|  | Junior doctors have to buy-in |
|  | Not all aware of study |
| **Trial staff roles** | Lacking confidence and motivation |
|  | Recruitment and trial paperwork demanding |
|  | Databases not user-friendly |
|  | Patient counselling and empathy |
|  | Staffing levels and support |
|  | Lack of confidence, familiarity |
| **Women presenting** | Out of hours |
|  | Infrequently |
|  | Not had 1st line or not had it for long enough |
| **Women ineligible** | Hadn't failed 1st line |
|  | Had study drugs |
|  | After amendment, had study drugs by IV |
|  | On anti-depressant or other |
|  | Language ability |
| **Women eligible** | Preferring previously known drug |
|  | Declining placebo |
|  | So unwell |
|  | Can't read and understand |
|  | A lot of much information |
|  | Needing a quick, definite solution |
|  | Preferring not to take medication because of possible risk |
|  | Perceived research burden |

**Table 3: Enablers for recruitment**

| **Topic** | **Enablers** |
| --- | --- |
| **Requirements of the clinical trial** | Adherence to RCOG guidelines |
|  | Study question was seen as relevant |
|  | Good staff relations and communication |
| **CTU** | Staff available and responsive |
|  | Good liaison |
| **Demands of a CTIMP protocol** | Counselling takes time, recruitment less so |
|  | GCP training was facilitated |
|  | Waiting time for eligibility |
|  | 12 hours to see if the IMP really does not work - was acceptable |
| **Publicity and education about the trial** | GPs |
|  | A&E staff |
|  | Clinical staff on duty and out of hours |
|  | Getting staff on board |
|  | Engaging non-clinical staff |
| **Trial staff roles** | Confidence and morale |
|  | Good teamwork and support for recruitment |
|  | Patient counselling and empathy |
|  | Staffing levels and workload |
|  | Confidence and familiarity |
| **Women presenting** | Out of hours |
|  | Frequently |
|  | Had 1st line |
| **Women ineligible** | Hadn't failed 1st line |
|  | On anti-depressants |
|  | Language ability |
| **Women eligible** | Counselling about the risks of a double blind trial |
|  | The experience of severe NVP |
|  | Reading and understanding information |
|  | Information |
|  | Want a quick, definite solution |
|  | Preferring not to take medication |
|  | Extra care and attention as research participants |
